# Composition and Medical Qualities of the Cartmel or Flookburgh Mineral Waters

**Published:** 1817-04

**Authors:** Robert Charnock


*For the London Medical and Physical Journal.*

*Composition and Medical Qualities of the Cartmel or Flookburgh Mineral Waters;* by ROBERT CHARNOCK, Esq.

SOME time ago you expressed a wish, in the Medical and Physical Journal, to know the Composition and Medical Qualities of the Cartmel or Flookburgh Mineral Water: if the following be worth a place in your valuable publication, it is at your service.

First, I shall endeavour to give you some idea from whence this far-famed water has its source; next, its analyzation and medical properties.

This water undergoes a variety of names, such as the Cartmel or Flookburgh Spa, but more properly the *Holly Well*, as those two villages are situated at a considerable distance from it. The water issues from a rock principally composed of lime-stone, whose perpendicular height is 300 feet, and its circumference about two miles; its juts extend into the bay of Morecamb, and, from its summit, which is clothed with rich verdure, there is an extensive and beautiful view of the bay and adjacent shore. The water flows through a small leaden tube, which emits one gallon in seventy seconds: the quantity of water was never known to receive any increase or diminution, notwithstanding the tide flows very near its outlet. The tide, at high water, extends a considerable way up each side of the rock. Near the Spa is a miserable fisherman's hut, (belonging to three gentlemen of very considerable fortune,) whose present resident has inhabited it near thirty years. For a small sum he supplies with water his numerous visitors, who generally reside at Flookburgh and Grange during the summer, a distance of two miles, where they find most excellent accommodations.

The component parts of this valuable and efficacious water, from a very accurate analysis, are as follow:

The water is transparent, and nearly free from smell. Its taste is saline, but not bitter or pungent.

It suffers no change by exposure to the air.

The specific gravity of the water, at 60° Fahrenheit, is 1·0048.

The

The following tests were successively applied, and presented the following appearances with the water:

1st. The gall test, turbid with a very slight precipitate.

2d. The lime-water had no effect.

3d. The colours of turmeric and violet were not sensibly affected.

4th. Muriate of barytes gave an immediate and abundant precipitate, not soluble in nitric acid.

5th. Solutions of silver or mercury afforded with the water dense and abundant precipitates.

6th. Oxalate of ammonia produced rather a copious precipitate.

7th. The water being freed from lime, pure ammonia had no sensible effect.

The abundant precipitates obtained by the nitrate of silver and barytic solutions, render it probable that the principal ingredients of this water are the muriate of sodæ and sulphate of lime: with these may be associated, in small quantities, the sulphate of magnesia, muriate of lime, and oxide of iron.

A pint of the water being evaporated, the solid contents were dried by a heat rather inferior to redness, and weighed 62 grains, which contain the following proportions, viz.

| | |
|---|---|
| Sulphate of lime ------ | $10\frac{6}{10}$ |
| ———— magnesia -- | $2\frac{4}{10}$ |
| Muriate of sodæ ------ | 49 |
| | 62 |

Before I knew the component parts of this water, I certainly supposed it contained more active ingredients; therefore its active and efficacious properties are not easily accounted for.

This mineral has been famed, from time immemorial, as a mild aperient and diuretic; and, when taken in large quantities, as a powerful purgative: it has long been the resort of the rich voluptuary, the poor mechanic, and the laborious miner; the bloated face it reduces to its original hue, and the miner returns with renovated health;—indeed it may be considered as a *panacea* for the workers in lead, for I have seen some of the Alstone moor miners, who had been at work for years before, come to drink this water the most emaciated objects that can be conceived, and, in the course of three or four weeks, nearly restored to their original health. Now, this effect is not induced in a common way, with drinking the water as a gentle aperient, but, on the contrary, with brisk and repeated purging. The water is drank in such a quantity every day for three or four

2 weeks weeks as to completely empty the bowels; or, if I may be allowed the expression, until not a particle of fæces remain. This may serve as a farther proof, if there needed any, of the vast utility of purgatives in restoring the secretions; for, instead of weakening the stomach and bowels, as we were once taught to believe, the contrary effect is induced—the digestive organs are restored, and muscular power increased.

The efficacy of this is not confined to obstructed chylopöietic viscera: it is of great service in cutaneous affections, proceeding from obstructed perspiration. I have also seen its good effects in cases of tenia and lumbricus. In a case of hydrothorax which came lately under my care, the patient had been drinking the water for a week, but without any material benefit. The digitalis was had recourse to, and the water drunk in such quantities as to produce four or five loose stools every day, with evident good effects; but the patient being under the necessity of leaving the country, I had not an opportunity of giving it a full trial. The same gentleman informed me, that, being in the same state three years ago, by drinking the water in large quantities, without the aid of any medicine, the effused fluid was evacuated, and he remained well for three years. I have no doubt that it assists the digitalis and other diuretics very materially, in stimulating the kidneys, and promoting absorption. When taken for the length of time I have mentioned, and then desisted from, the system does not seem to require it, and no astringent effect is induced from its long continuance. I don't think that its purgative quality is very much quickened by any purgative taken previously: it appears to have, in some measure, its own specific action. It may be taken as an alterative, from half to a whole pint every morning; a little warm when taken as a purgative *ad libitum*.

I am afraid, gentlemen, I have trespassed too much on your time; but the subject deserves every notice, and I wish it had fallen into hands that would have done it more justice,—for the Holly-Well water deserves to be better known, to make it more generally useful.

*Cartmel; Oct.* 21, 1816.

---

When we consider the *mille mali species*, it is impossible, if we admit an all-designing Providence, not to admit also *mille salutis*. On this account, instead of considering our Pharmacopœia as too abundant in *materia medica*, we have always wished it more copious. As Mr. Charnock very modestly observes, before he made his analysis, he conceived the waters must have contained more potent principles than he could discover. This, however, may only arise from the imperfection of our chemistry. Whilst we we are on this subject, we would take the liberty of proposing an additional test or agent in the analysis of every substance intended for medicine. Why should not gastric juice be used, and afterwards bile? The former, we know, dissolves what no chemical substance is capable of; and, if we learn little by either as to the properties of the subject of examination, we may gradually learn more of the fluids themselves. We cannot conclude these remarks without expressing a wish that these waters had the advantage of many others, probably less efficacious—good society, and good accommodations nearer than two miles from the spring. —We can hardly admit the apology of our correspondent relative to the inaccuracy of his copy. He must be aware how much more easily it might have been corrected by himself than by us. We are still doubtful of the accuracy of punctuation in the last line of the last paragraph but one.—EDIT.

---

*For the London Medical and Physical Journal.*

